# Safety and Effectiveness of Mycophenolate in Systemic Sclerosis. A Systematic Review

**DOI:** 10.1371/journal.pone.0124205

**Published:** 2015-05-01

**Authors:** Mohammed A. Omair, Abdulaziz Alahmadi, Sindhu R. Johnson

**Affiliations:** 1 Toronto Scleroderma Program, Division of Rheumatology, Department of Medicine, Toronto Western Hospital, Mount Sinai Hospital, Toronto, Ontario, Canada; 2 Institute of Health Policy, Management and Evaluation, University of Toronto, Toronto, Ontario, Canada; 3 Division of Rheumatology, Department of Medicine, King Saud University, Riyadh, Saudi Arabia; Nippon Medical School Graduate School of Medicine, JAPAN

## Abstract

**Background:**

Mycophenolate is increasingly being used in the rheumatic diseases. Its main adverse effects are gastrointestinal, myelosuppression, and infection. These may limit use in systemic sclerosis (SSc) since gastrointestinal involvement is common. The objective of this study is to evaluate gastrointestinal adverse events of mycophenolate in SSc. Secondarily we evaluated other adverse events, and the effectiveness of mycophenolate in skin and lung disease.

**Methods:**

A literature search of Medline, Embase, Cochrane Central Register of Controlled Trials, and CINAHL (inception-2013) was performed. Studies reporting use of mycophenolate in SSc patients, adverse events, modified Rodnan skin score (MRSS), forced vital capacity (FVC), or diffusing capacity of carbon monoxide (DLCO) were included. The primary outcome was gastrointestinal events occurring after the initiation of mycophenolate. Secondary safety outcomes included myelosuppression, infection, malignancy, and death after the initiation of mycophenolate.

**Results:**

617 citations were identified and 21 studies were included. 487 patients were exposed to mycophenolate. The mean disease duration ranged between 0.8-14.1 years. There were 18 deaths and 90 non-lethal adverse events. The non-lethal adverse events included 43 (47.7%) gastrointestinal events, 34 (26%) infections, 6 (5%) cytopenias and 2 (2%) malignancies. The most common gastrointestinal events included diarrhea (n=18 (14%)), nausea (n=12 (9%)), and abdominal pain (n=3 (2%)). The rate of discontinuation ranged between 8%-40%. Seven observational studies reported improvement or stabilization in FVC, and 5 studies report stabilization or improvement in MRSS.

**Conclusion:**

Mycophenolate-associated gastrointestinal adverse events are common in SSc, but not severe enough to preclude its use. Observational data suggests mycophenolate may be effective in improving or stabilizing interstitial lung disease, and skin involvement.

## Introduction

Systemic Sclerosis (SSc) is a systemic rheumatic disease characterized by extracellular collagen deposition, fibrosis and altered endothelial function. Abnormalities in both T and B cells play an important role in the pathogenesis of SSc.[1] The presence of specific autoantibodies that are present at the onset of the disease is indicative of a pathogenic role.[2] These findings have been the background of many trials of biologic and non-biologic disease modifying agents in SSc.[3,4] Mycophenolate mofetil is a prodrug of mycophenolic acid (MPA), an inhibitor of inosine monophosphate dehydrogenase[3,4], an enzyme involved in the synthesis of guanosine nucleotides.[5] T and B lymphocytes are dependent on this pathway, resulting in immunosuppressive effects of mycophenolate preparations.[5] MPA has been also found to reduce chronic allograft nephropathy and interstitial fibrosis by inhibiting transforming growth factor β[6,7] which has been recognized as an important molecule in the pathogenesis of SSc and other fibro-proliferative diseases.[8] Its clinical efficacy, safety profile, pharmacokinetics and pharmacodynamics properties made it a standard of care in solid organ transplantation and lupus nephritis.[9,10] The main side effects observed are gastrointestinal disturbance, myelosuppression, and increase risk of infection. Compared with mycophenolate mofetil, enteric-coated mycophenolate sodium has delayed gastrointestinal absorption, thereby potentially reducing gastrointestinal adverse events.[11] Its coating dissolves at pH >5, thereby facilitating small intestine delivery.[12]

Gastrointestinal side effects are dose dependent in patients treated with mycophenolate and include nausea, vomiting, abdominal pain, diarrhea and rarely gastrointestinal bleeding and perforation. Mycophenolate discontinuation or dose reduction is needed in 40% to 50% of transplant patients which is associated with increased graft loss.[13] This maybe a limitation of its use in SSc patients since gastrointestinal involvement is very common.[14] Gastrointestinal involvement adversely affects the quality of life of SSc patients.[15,16] Treatment is usually symptomatic with limited effectiveness in advanced cases.[14,17] Thus, clinicians are left with a dilemma. Mycophenolate may have beneficial effects in SSc patients, however the adverse impact on the gastrointestinal system may not warrant its use. A systematic review and meta-analysis of mycophenolate in SSc related interstitial lung disease conducted between 2006–2011 reported clinically significant infection, leucopenia, and elevated liver enzymes; but did not report detailed gastrointestinal adverse events.[18] The objective of this study was to evaluate gastrointestinal adverse events of mycophenolate in SSc. Secondarily we evaluated the other adverse events and the effectiveness of mycophenolate in treating SSc skin and lung disease.

## Materials and Methods

### Literature search

A systematic review of the literature was conducted through the University Health Network (UHN) library with the assistance of an information specialist. Databases included Ovid MEDLINE(R), Embase, Cochrane Central Register of Controlled Trials, Cochrane Database of Systematic Reviews, and CINAHL (all inception-2013).

The following keywords with mapping of term to subject headings were used in the database search: (systemic scleroderma or systemic sclerosis or diffuse scleroderma) and (mycophenolate mofetil or mycophenolate sodium or mycophenolic acid or inosine monophosphate dehydrogenase or cellcept or myfortic). The search was restricted to humans, but no language restriction was applied. ChemID plus was used to identify other terms for mycophenolate. The bibliographies of included studies and reviews were searched.

### Study Selection

Abstracts were reviewed to identify studies that described the use of mycophenolate in SSc patients. Inclusion criteria included 1) peer reviewed observational studies and randomized trials, 2) report use of at least one dose of MMF or MS as an exposure, 3) report of efficacy or effectiveness outcomes, or adverse events, 4) age ≥18 years. Machine translation software was used to translate non-English language articles. Efficacy analysis included only prospective and retrospective studies with 10 or more patients.

### Data abstraction

Two investigators (MO, AA) independently reviewed the title and abstract of each citation and applied the inclusion and exclusion criteria to select studies for full review. A standardized data abstraction form was used to collect study design, sex, age, SSc disease duration, SSc subtype, autoantibodies, organ involvement, and medication. The reviewers were blinded to the names of journals, authors, and institutions when performing data abstraction.

### Outcomes

The primary outcome was gastrointestinal events occurring after the initiation of mycophenolate or worsening after treatment exposure. This included nausea, vomiting, bloating, abdominal pain, diarrhea, upper and lower GI bleeding. Secondary safety outcomes included myelosuppression, infection, malignancy, rate of discontinuation with reason (adverse event or failure), and death occurring after the initiation of mycophenolate.

Secondary effectiveness outcomes included the modified Rodnan skin score (MRSS), total joint count, swollen joint count, tender joint count, forced vital capacity (FVC), diffusing capacity of carbon monoxide (DLCO), vital capacity (VC) and changes on high resolution CT scan. All outcomes were evaluated at 3, 6, 9 and 12 months after exposure to mycophenolate. Outcomes were collected using a standardized data collection form by 2 independent abstractors. Disagreements were resolved by consensus or a 3^rd^ party (SRJ), if needed.

### Exposure

The exposure was defined as treatment with any preparation of mycophenolate. No minimum dose or duration of treatment was pre-specified.

### Analysis

Descriptive statistics were used to summarize the data. All analyses were conducted using RStudio. The systematic review conforms with the PRISMA guidelines (S1 Table).

## Results

### Patients

**Fig 1:**
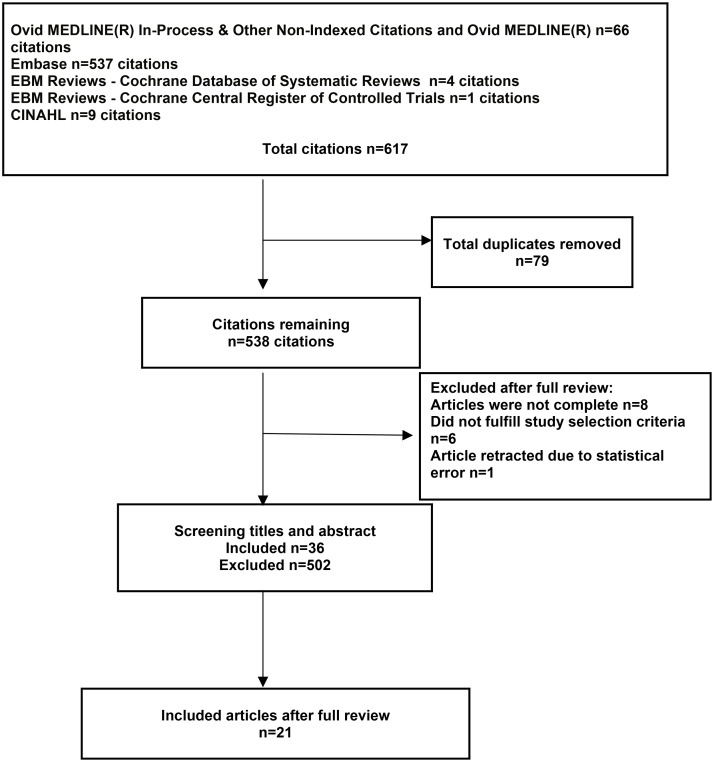
Systematic review flow diagram.

Six hundred and seventeen citations were identified, of which 21 fulfilled the criteria for inclusion in the analysis. (Fig 1) The clinical and serologic characteristics of patients are summarized in Table 1. Study designs included prospective cohort (n = 7)[19–25], retrospective cohort (n = 6)[26–31], case-control (n = 1)[32] and case reports/series (n = 7)[33–38]. Five studies compared mycophenolate mofetil to placebo or to other treatment modalities[23,27,31–33]. A total of 487 patients have been reported to be exposed to mycophenolate, with the proportion of females ranging between 50–100%. The mean disease duration ranged between 0.8–14.1 years.

**Table 1:** Clinical and serological characteristics of systemic sclerosis patients.

Author	Design	Controls	n	Age mean/median (years)	Disease duration mean/median (years)	Female n(%)	Diffuse n(%)	SCL70 n(%)	ACA n(%)	Other
**Stratton et al.[19]**	P	No	13	NA/52	NA/0.75	10 (77)	13(100)	2(15)	0	U1 RNP 2(15) U3 RNP 1(8) RNA PIII 2(15)
**Vanthuyne et al.[25]**	P	No	16	47/NA	0.8/NA	12 (75)	13(81)	NA	NA	NA
**Nihtyanova et al.[27]**	R	Yes	109	NA/NA	NA	90(83)	101(93)	35(32.1)	2(1.8)	U3 RNP 5(4.6) RNA PIII 26(23.9)
**Le et al.[31]**	R	Yes	98	48.4/NA	1.83/NA	81 (83)	98(100)	24(24)	2(2)	NA
**Mendoza et al.[20]**	P	No	25	48.6/NA	14.1/NA	NA	25(100)	9(36)	0	NA
**Cuomo et al.[36]**	C	No	1	63/NA	7/NA	1 (100)	1(100)	NA	NA	NA
**Saketkoo et al.[37]**	C	No	4	50.5/55.5	6.25/6	4 (100)	NA	NA	NA	NA
**Zamora et al.[28]**	R	No	17	50.8/NA	NA/2	10 (59)	15(88)	NA	NA	NA
**Gerbino et al.[26]**	R	No	13	NA/52	NA/5	8 (62)	9(69)	4(31)	Na	NA
**Derk et al.[21]**	P	No	15	50/NA	1.1/NA	10 (66.7)	15(100)	6(40)	0	NA
**Koutroumpas et al.[29]**	R	No	10	59.7/NA	7.7/NA	8 (80)	10(100)	10(100)	0	NA
**Simeón-Aznar et al.[22]**	P	No	14	NA/54.4	NA/6.5	13 (93)	8(57)	8(57)	1(7)	NA
**Liossis et al.[24]**	P	No	6	46/NA	3.4/NA	4 (66.7)	6(100)	6(100)	0	NA
**Plastiras et al.[33]**	C	Yes	7	58/NA	NA	6 (86)	NA	Na	Na	NA
**Busquets et al.[34]**	C	No	1	NA/39	NA/0.67	NA	1(100)	NA	NA	NA
**Bandelier et al.[39]**	C	No	1	63/NA	4/NA	1 (100)	1(100)	NA	NA	NA
**Bérezné et al.[30]**	R	No	5	NA/NA	NA	NA	NA	NA	NA	NA
**Gonzalez-Nieto et al.[43]**	C	No	5	NA/NA	NA	NA	NA	NA	Na	NA
**Gulamhusein et al.[38]**	C	No	2	52/NA	NA	1 (50)	2(100)	Na	Na	NA
**Herrick et al.[23]**										
**Protocol 1**	P	Yes	29	NA/55.1	NA	18 (62)	29(100)	8(33)	NA	RNA PIII 1(8)
**Protocol 2**	P	Yes	25	NA/52.7	NA	20 (80)	25(100)	5(20)	NA	RNA PIII 1(4)
**Protocol 3**	P	Yes	61		NA	44(72)	61(100)	14(24)	NA	RNA PIII 9(22)
**Panopoulos et al.[32]**	CC	Yes	26	48/NA	5.8	24 (92)	18 (69)	19 (73)	NA	NA

P: Prospective, R: Retrospective, C: Case report/series, CC Case-control, ACA: Anti-centromere antibody, RNA PIII: RNA polymerase III, CAU: Caucasians, AA: African American, CAR: Caribbean, NA: Not available

### Mycophenolate use

Mycophenolate preparations vary across studies (mycophenolate mofetil n = 19, mycophenolate sodium n = 1, both n = 1). Mycophenolate was used as a first line agent in 3 studies[20,28,39]. Mycophenolate compounds were used for induction (n = 13 studies[20–22,24,26–29,34,37,39–41]), for maintenance (n = 18 studies[19–22,24–29,33,34,37–42]), for both induction and maintenance (n = 14 studies[20–22,24–29,34,36,37,39,41]), and as rescue therapy (n = 2 studies[30,43]). Herrick et al.[23] reported a prospective observational study which assessed 5 immunosuppressive regimens: intravenous cyclophosphamide followed by mycophenolate mofetil; anti-thymocyte globulin (ATG) followed by mycophenolate mofetil; mycophenolate mofetil alone; and other immunosuppressant treatment; and no disease-modifying treatment. Other induction regimens were ATG and glucocorticosteroids combination[19], and cyclophosphamide[30,33,43]. Prior immunosuppression was reported in 12 studies.[22–27,30,31,33,36,37,43] Concomitant glucocorticosteroids were used in 11 studies[19,21–25,29,37–39,41]. Treatment of skin disease and lung involvement were the only 2 indications. In most of the studies the target dose was 2g/day. This dose was achieved in 42%-88% of the time.[20,21,27,41]

### Safety

**Fig 2:**
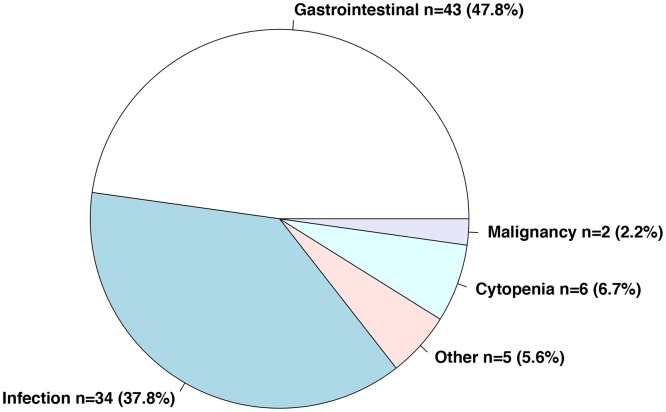
Circle chart of non-lethal adverse events in SSc patients treated with mycophenolate.

**Fig 3:**
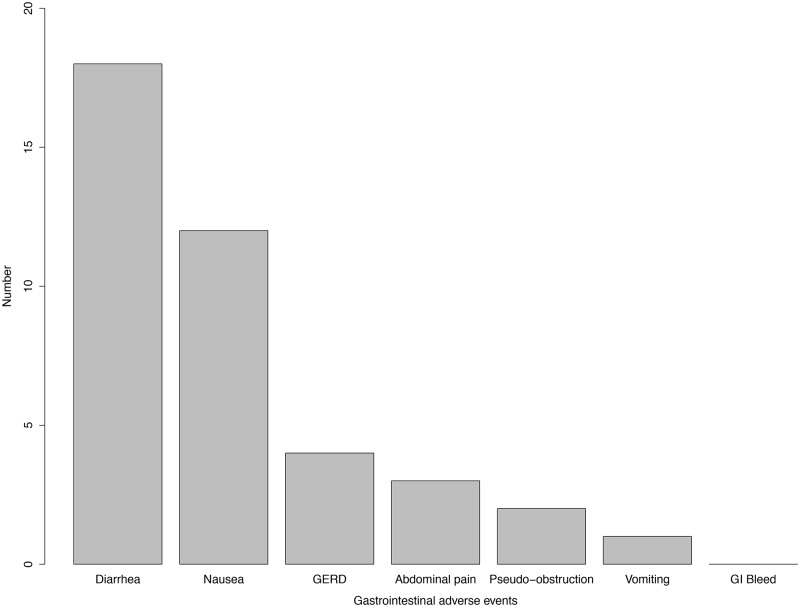
Bar graph illustrating gastrointestinal adverse events in SSc patients treated with mycophenolate.

There were 18 deaths and 90 non-lethal adverse events. The non-lethal adverse events included 43 (47.7%) gastrointestinal events, 34 (26%) infections, 6 (5%) cytopenias and 2 (2%) malignancies. (Fig 2) The most commonly reported gastrointestinal events included diarrhea (n = 18 (14%)), nausea (n = 12 (9%)), gastro-esophageal reflux disease (GERD) (n = 3 (3%)), abdominal pain (n = 3 (2%)), pseudo-obstruction (n = 2 (2%)), and vomiting (n = 1 (0.8%)). There were no reports of gastrointestinal bleeding. (Fig 3) The most commonly reported non-gastrointestinal adverse events were infections (n = 34 (26%), cytopenias (n = 6 (5%)) and malignancy (n = 2 (2%)). Sites of infection included the respiratory tract (n = 11), urinary tract (n = 2), skin (n = 4), eye (n = 1). The site of infection was not specified for 15 cases. Respiratory infections included the upper respiratory tract (n = 3), bronchus (n = 4), bacterial pneumonia (n = 3) and aspergillus (n = 1). Cytopenias included lymphopenia (n = 2), neutropenia (n = 1), anemia (n = 2) and not specified (n = 1).

Eighteen (14%) mycophenolate treated patients died. The cause of death was clearly described in 1 patient (dilated cardiomyopathy).[20] In the study by Herrick et al. 23 death occurred, 13 (57%) of whom were in the mycophenolate groups.[23] The cause of death was identified in 17 of these patients but not attributed to a specific treatment. The mycophenolate monotherapy group had significantly better survival than other treatment groups. This was replicated by Nihtyanova et al. who reported a 5-year survival of 91.7% in mycophenolate treated patients compared to 77.8% in the control group (p = 0.01).[27] Fifty-two (10.9%) patients discontinued mycophenolate. The reported rate of discontinuation ranged between 8%-40%. Nihtyanova et al. reported a retention rate in the mycophenolate group to be 79%, 59% and 66% at 1-year, 1–3 years and 5-years, respectively.[27] Mendoza et al. reported that 72% of their patients were still on mycophenolate at 1 year.[20]

### Effectiveness

#### Skin

The treatment duration ranged from 3–60 months. Eight studies reported values of the MRSS.[19–21,23,25,27,29,41] Table 2. The mean and the median baseline MRSS ranged from 17.2–28 and 21.5–32, respectively. Stratton et al. evaluated mycophenolate as maintenance therapy in 13 patients with <2 years disease duration after receiving induction ATG for 5 days.[19] The MRSS improved from 28 to 17, and the finger to palm distance at 3 and 6 months (p<0.05) improved, but not at 12 months. Vanthuyne et al. treated 9 patients with only skin involvement and disease duration less than 3 years with mycophenolate and IV methylprednisolone for 3 days and then monthly for 5 months, in addition to daily low dose glucocorticosteroids.[25] Fifty-six patients with only skin disease responded at 12 months compared to 69% of the total study patients. Le et al. evaluated mycophenolate effects on MRSS diffuse SSc patients in the Johns Hopkins Scleroderma Center with patients from 3 different clinical trials (D-penicillamine, recombinant human relaxin and bovine type I collagen).[31] The change in the MRSS was not superior to the human relaxin study at 6 months (p = 0.059), but was statistically significantly lower than D-penicillamine study (p<0.001), and bovine type I collagen study (p = 0.002) at 12 months.[31] A subgroup analysis suggested that the change in the MRSS in the MMF combined with other immunosuppressive (methotrexate or IV immunoglobulins) agent group was greater than mycophenolate monotherapy (-0.83±7.6 versus -4.64±6.2; p = 0.047). The MRSS mean change between groups at 12 months was not statistically different (-7.73±13.5 versus −10.14±8.6; p = 0.496).[31] Mendoza et al. studied the effect of mycophenolate mofetil on MRSS and the extent of body surface area (BSA) affected assessed by the rule of nines using the burns-victim diagram.[20] It was noticed that both the MRSS and BSA progressed early in the study reaching a peak at 3–6 months. The MRSS improved from 24.56±8.62 to 14.52±10.9 (p = 0.004) and affected BSA decreased from 36%±16% to 14%±13.3% (p = 0.00001). Additionally, effect of therapy was assessed on pre and post skin biopsies in 3 patients. Post-treatment biopsy revealed decrease in the abundance and thickness of collagen bundles and in their compact appearance in the dermis with reappearance of hair follicles and sweat and sebaceous glands. Also, RNA was extracted from all the biopsies and expression of fibrosis-related genes was evaluated. Treatment resulted in reduction in the expression of *COL1A1*, *COL1A2*, *COL 4A1*, *COL 11A1 COL 14A1*, *CTGF*, *FN1*, *ACTA2*, and *TGFB1*.

**Table 2:** Effect of mycophenolate mofetil on modified Rodnan skin score.

Author	Duration of Therapy (months)	Mean baseline MRSS	Median baseline MRSS	MRSS at end of study	Level of significance
**Stratton et al.[19]**	12	28	NA	17	p < 0.001
**Vanthuyne et al.[25]**	12	20	NA	13	p < 0.0001 for all patients p = 0.002 for skin group
**Nihtyanova et al.[27]**	60	NA	26	11	NA
**Le et al.[31]**	12	24.4	NA	17.5	p < 0.001
**Mendoza et al.[20]**	Mean 18.2	24.56	NA	14.5	p = 0.0004
**Derk et al.[21]**	12	22.5	21.5	8.4	p < 0.0001
**Koutroumpas et al.[29]**	12	17.2	NA	17.7	p = 0.55
**Herrick et al.[23]**					p = 0.43
**Protocol 1**	36	NA	24	NA	–1.81 (95%CI—4.08, 0.460)
**Protocol 2**	36	NA	32	NA	–4.46 (95%CI—6.69, –2.23)
**Protocol 3**	36	NA	23.5	NA	–3.10 (95%CI—4.27, –1.93)

#### Lung

Thirteen studies reported pulmonary function tests at baseline.[19–21,23–26,28,29,32,37,41] Significant reductions in the level of DLCO occurred in 2 studies[24,25], but change in the remaining studies was not significant.[19–21,26,28,29,41] Only the study by Koutroumpas et al. reported significant improvement in FVC.[29] Vanthuyne et al. reported a significant improvement in both the predicted and the forced expiratory volume at 1 second (FEV_1_) from 76% to 90% (p = 0.003) and 1971ml to 2347ml (p = 0.0009), and in VC from 2598 ml to 2943 ml (p = 0.012) but not the predicted VC (p = 0.099).[25] Additionally the 6 minute walk increased from 506 meters to 567 meters, but not reaching significance (p = 0.011). Derk et al. reported a non-significant 7% increase in the TLC from 100.2% to 107.2%,[21] while Mendoza et al. reported a non-significant reduction of TLC from 89.5% to 85.3% (p = 0.13).[20] In this subgroup of patients only 3 patients had a 10% or more reduction of the predicted TLC. Simeon-Aznar et al. report the use of mycophenolate sodium in 14 SSc patients with interstitial lung disease. There was no change in median FVC, FEV1 and DLCO at 12 months compared to baseline, suggesting mycophenolate sodium prevents worsening.[22] Table 3.

**Table 3:** Effect of mycophenolate on pulmonary function.

	Duration of Therapy (months)	Baseline DLCO (% predicted)	DLCO at end of study (% predicted)	Level of significance	Baseline FVC (% predicted)	FVC at end of study (% predicted)	Level of significance
**Stratton et al.[19]**	12	66	63	Not significant	87	88	Not significant
**Vanthuyne et al.[25]**	12	63	76	p = 0.0009	NA	NA	NA
**Le et al.[31]**	12	77.4	79.2	p = 0.336	79.4	80.7	p = 0.264
**Mendoza et al.[20]**	18.2	69	70.5	p = 0.45	NA	NA	NA
**Cuomo et al.[36]**	5	60	NA	NA	104	NA	NA
**Saketkoo et al.[37]**	3	30	NA	NA	80	NA	NA
**Zamora et al.[28]**	24	50	NA	p = 0.84	72	NA	p = 0.57
**Gerbino et al.[26]**	24	51	NA	p = 0.38	NA	NA	NA
**Derk et al.[21]**	12	71.2	74.3	Not significant	99.2	105	Not significant
**Koutroumpas et al.[29]**	12	80.7	86.7	p = 0.66	79.5	87.1	p = 0.04
**Simeon-Aznar et al.[22]**	12	40	37	NA	64	64	NA
**Liossis et al.[24]**	4–6	64.2	75.4	p = 0.033	65.6		p = 0.057
**Herrick et al.[23]**							
** Protocol 1**	36	58.8	NA	NA	76	NA	NA
** Protocol 2**	36	76.1	NA	NA	93.3	NA	NA
** Protocol 3**	36	71.5	NA	NA	87.8	NA	NA

NA not available, DLCO diffusing capacity of carbon monoxide, FVC functional vital capacity, VC vial capacity, TLC total lung capacity

CT findings were evaluated in 6 studies.[24–26,28,32,37] Vanthuyne et al. evaluated HRCT by a semi quantitative score, which consists of ground-glass opacity, consolidation areas, interlobular thickening and honeycombing on 5 predefined HRCT sections (aortic arch, azygos arch, distal portion of the bronchus intermedius, right inferior pulmonary vein and liver dome).[25] Grading was from 0 to 4 (0 = normal; 1 = 25% surface involvement; 2 = 26–50% involvement; 3 = 51–75% involvement; 4 = 76–100% involvement). The radiological scores correspond to the mean (± SD) of the gradings made on the 5 HRCT sections. Except for stabilization of the interlobular thickening, all scores improved at the end of study but not reaching statistical significance. Saketkoo et al. reported the effect of mycophenolate mofetil ion 10 patients with different connective tissue disease including 4 SSc patients.[37] HRCT improved in 1 patient, stabilized in 2 patients, and not reported in 1 patient. These findings were consistent with PFT results. Zamora et al. reported subjective assessment of HRCT by 2 radiologists in 15 patients, 11 of whom had nonspecific interstitial pneumonitis, and 4 had usual interstitial pneumonitis.[28] The HRCT findings improved (n = 1 (7%)), remained stable (n = 11 (73%), and progressed (n = 3 (20%)). Gerbino et al. reported 13 patients with baseline HRCT.[26] The most common baseline imaging finding was ground glass appearance with or without reticular opacities (92%). They reported assessment of 6 patients with serial HRCT.[26] Three of six improved, while the other 3 remained stable. Liossis et al. reported resolution of ground glass appearance on the HRCT of 4 of 5 patients with early disease.[24] Nihtyanova et al. report a retrospective cohort study where diffuse SSc patients on mycophenolate mofetil were compared to patients on other immunosuppressive therapies.[27] At baseline, a greater proportion of the control group had pulmonary fibrosis (14.3%) compared with mycophenolate mofetil exposed patients (7.3%). Twelve percent of the mycophenolate mofetil group developed pulmonary fibrosis compared with 19% of the control group (p = 0.037) over 5 years. Moreover, a significant better 5-year survival was also identified in the MMF group treated both from disease onset (95.4% versus 85.7%, p = 0.027) and from treatment initiation (91.7% versus 77.8%, p = 0.012).[27] One study reported worsening CT findings in the mycophenolate exposed patients compared to the cyclophosphamide exposed patients.[32]

#### Quality of Life and Disability

Quality of life (QoL) and disability were reported in 4 studies.[19,21,25,41] Stratton et al. report the reduction in the MRSS and finger to palm distance did not lead to a significant improvement in the patient global assessment, Euro-Qol score or the SSc functional assessment score.[19] Vanthuyne et al. analyzed each component of the scleroderma health assessment questionnaire (SHAQ).[25] Only the HAQ-DI (p = 0.021) and the pain visual analog scale (p = 0.031) significantly improved. The GI component of the SHAQ improved from 26 to 20 (p = 0.804). Le et al. reported that compared with baseline, the HAQ-DI score improved after 12 months (1.1±0.6 versus 0.94±0.7; p < 0.001).[31] Derk et al. reported that the mean SF-36 improved (65.9 to 77.6) p = 0.05.[21] When the 2 components were analyzed separately, only the physical component increased significantly (p = 0.05) compared to the mental health component (p = 0.15).[21]

#### Other Outcome Measures

The Medsger Severity Score (MSS) includes nine categories (general, peripheral vascular, skin, joints, muscular, gastrointestinal, pulmonary, cardiac and renal) of SSc organ involvement, which are rated on a scale of 0–4, where a higher the number indicates more severe organ involvement.[44] The MSS was reported in 4 studies. Le et al. reported that after 12 months of mycophenolate mofetil therapy the general (p = 0.013) and muscle severity scores (p = 0.003) improved, whereas the cardiac (p = 0.655), pulmonary (p = 0.490), peripheral vascular scores (p = 0.061), renal (p = 0.317), and joint scores (p = 0.103) did not change significantly.[31] They also report gastrointestinal severity score (p = 0.025) worsening. Mendoza et al. reported only improvement in the skin index (2.2±0.71 versus 1.52±0.77, p = 0.0003).[20] Derk et al. reported significant improvement occurred in the general (p = 0.05), peripheral vascular (p = 0.04) and skin (p = 0.0003), while the GI score improved from 0.33 to 0 but not reaching statistical significance (p = 0.08).[21] None of the studies reported the effect of mycophenolate on total joint count, swollen or tender joint counts.

## Discussion

This systematic review suggests that gastrointestinal adverse events are common in SSc patients treated with mycophenolate. Almost a half of adverse event were gastrointestinal including diarrhea, nausea, vomiting, pseudoobstruction and abdominal pain. The most commonly reported non-gastrointestinal adverse events were infections and cytopenias. The reported rate of discontinuation ranged between 8–40%. However mycophenolate appears to be effective in improving or stabilizing interstitial lung disease, and may be effective for skin involvement.

Despite the frequent worsening of GI symptoms after use of mycophenolate, it appears to be a reasonably tolerated therapeutic option. The etiopathogenesis behind gastrointestinal adverse events could be due to the effect on enterocytes that are 50% dependent on the purine synthesis pathway that is blocked by mycophenolate. As a result, mucosal erythema, excess fluid secretion, gastric and small intestine ulceration occur. In addition, intestinal invasion by common pathogenic and opportunistic organism may occur, especially in the presence of leucopenia.[45] Interestingly, there were no reports of gastrointestinal bleeding. The rate of gastrointestinal adverse events in SSc patients is at least similar, if not less frequent, than renal transplant patients.[46] It should be noted however, that reporting of gastrointestinal adverse events was subjective in most of the studies. The UCLA Scleroderma Clinical Trial Consortium Gastrointestinal Tract Instrument (UCLA-SCTC GIT) 2.0 is a validated tool that is capable of assessing the entire GI tract and can be used to assess the impact of mycophenolate on the GI tract in a more objective manner.[47]

With regards to other adverse events, the rate of infection was low compared to patients with SLE in clinical trials evaluating mycophenolate mofetil and mycophenolate sodium.[48–50] We also observed that the rate of discontinuation of mycophenolate in SSc patients due to adverse events was quite wide. However, it should be noted that the studies we report in this systematic review were not clinical trials, so it may be that adverse events were not collected systematically and are likely underreported.

Mycophenolate mofetil appears to have a beneficial effect of skin, interstitial lung disease and survival. The observed reduction in the MRSS and the MSS skin index is more than expected from the natural history of the disease. In studies evaluating mycophenolate mofetil in SSc associated interstitial lung disease, respiratory symptoms, lung physiology and radiological changes improved or at least stabilized. This suggests that aiming at stabilization of lung function would be a reasonable goal. Although speculative is seems rational that MMF represents a safe and relatively efficacious immunomodulatory agent for maintenance treatment in patients with SSc. Nevertheless, results from Scleroderma Lung Study II are greatly anticipated to shed further light on that issue. The promising results of mycophenolate compounds in SSc patients may be related to their anti-fibrotic effect through inhibiting TGF-β and fibroblast proliferation through IMPDH-dependent and IMPDH-independent pathways.[51,52] Additionally they have been shown to inhibit collagen deposition.[53]

A limitation to our study is that heterogeneity in study design and sample selection prevented us from aggregating all the data in a meta-analysis. Furthermore, all of the studies involved non-random allocation of exposure to mycophenolate, thereby potentially introducing bias. It should be noted that the majority of patients treated with mycophenolate had previously received cytotoxic treatment or were also under corticosteroid or other immunosuppressant treatment while on mycophenolate therapy. It is possible that the previous or co-interventions account for some of the reported side-effects and/or beneficial effects that are attributed to mycophenolate use. None of the published studies reported the effect of mycophenolate on total or swollen joint counts, so we were unable to comment on the effectiveness of mycophenolate on inflammatory arthritis in SSc. Other reports have shown that joint counts are infrequently reported outcomes in SSc studies.[54,55] This systematic review does synthesize the known literature, and is the first to explicitly synthesize the safety profile. Mycophenolate sodium is thought to have less gastrointestinal adverse events than mycophenolate mofetil. However, there was insufficient evidence in the published literature to evaluate if this hypothesis is true in SSc.

## Conclusion

Gastrointestinal adverse effects are common in SSc patients treated with mycophenolate, but are usually not severe enough to preclude its use. Observational data suggests mycophenolate is a safe and may be an effective therapeutic modality with a beneficial effect on skin thickening and progression of lung involvement. Randomized controlled trials evaluating mycophenolate in SSc patients, including the Scleroderma Lung Study II, are needed to confirm these findings.

## Supporting Information

S1 Table PRISMA checklist. (DOCX)
